# How can you trust what you read?

**DOI:** 10.1039/d3sc90068a

**Published:** 2023-04-21

**Authors:** May C. Copsey, Andrew I. Cooper

## Abstract

*Chemical Science* is introducing the option of transparent peer-review for authors. Editor-in-Chief Andy Cooper and Executive Editor May Copsey take a look at the reasons why, and how this will work.
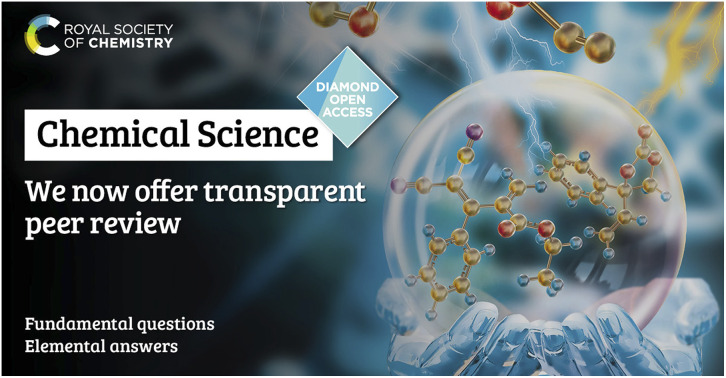

## Introducing transparent peer-review

Trust is at the heart of scholarly publication and peer review is an established mechanism for deciding which articles make it through to publication. However, confidence in peer review has been shaken recently, as witnessed by the increasing number of articles retracted due to fake or ‘rigged’ peer review.^1^

The Royal Society of Chemistry supports the principles of open science, which include working towards a more open and transparent research culture.^2^ This means that, in addition to our commitment to achieve 100% open access within five years, we will be working with our community to implement agreed best practice in open science across our journals.

At *Chemical Science* we are committed to embracing open science and we believe that the publishing process should be as transparent as possible. We believe in the power of open access to disseminate research more widely, and for some time now, we have made *Chemical Science* diamond open access (OA with no article-processing charges) to allow all authors to openly share their work.

We are now taking the next natural step to shed more light on what happens during the peer review process. Through this and other initiatives, we hope to rebuild trust in peer review as a valuable and constructive part of the publication process.

## Ever read a paper and wondered what the reviewers and editors thought?

From now on, *Chemical Science* will give authors the option to choose whether they want the anonymous reviewers’ comments, editor’s decision letter, and their own response to be published alongside their published article.

While there are many different definitions, this is what we mean by transparent peer review. In our implementation, reviewer comments will remain anonymous unless the individual reviewer chooses to sign their report. This gives both authors and reviewers a choice and it takes on board, for example, concerns raised by some early career researchers about compulsory signing of reviewer reports. The decision to introduce this particular mechanism in *Chemical Science* follows successful experimentation with transparent peer review in several other RSC journals, where we have seen an encouraging number of authors choosing this option.

By enabling readers to read the discussions between authors, reviewers and editors, we hope to provide an additional level of assurance in the peer review process. This will allow researchers to see how decisions have been reached, what information was used to inform that decision and, ultimately, why something was accepted.

By being more transparent about this decision-making process, we hope to build trust and showcase the fair, rigorous and inclusive peer review that we strive to deliver. In turn, this extra level of scrutiny will help us to ensure research integrity and reproducibility.

We believe that transparent peer review also has a role in highlighting the excellent contributions of our reviewers and editors, as well as providing future guidance and models for those new to peer review, such as early career researchers. It may also encourage higher-quality and more constructive reviewer comments.

Authors have no obligation to take part and can opt out at any decision stage throughout the process. Reviewers will be informed that if the author selects transparent peer review their anonymous comments will be published under a creative commons licence. They will need to confirm their agreement to this *via* a specific question in the reviewer report form.

More information and support on transparent peer review can be found in the FAQs on our website.^3^

We look forward to seeing the results of this new option in *Chemical Science* as we work toward a more open science culture.

Andy Cooper, Editor-in-Chief, *Chemical Science*, & Department of Chemistry, University of Liverpool, UK

May Copsey, Executive Editor, *Chemical Science*

## Supplementary Material

SC-014-D3SC90068A-s001
