# Supplementary material for: How can you trust what you read?
Source: Chem Sci. 2023 Apr 21;14(17):4447–8. doi: 10.1039/d3sc90068a (PMC10155907; doi:10.1039/d3sc90068a)
Supplement: SC-014-D3SC90068A-s001 [file SC-014-D3SC90068A-s001.pdf]

Electronic Supplementary Material (ESI) for Chemical Science.
This journal is © The Royal Society of Chemistry 2023

# Frequently Asked Questions on Transparent Peer Review

## What is Transparent Peer Review?

Transparent peer review is where the editor's decision letter, reviewers' comments and authors' response for all versions of the manuscript are published alongside the article under an Open Access Creative Commons licence (CC-BY).

## Why offer Transparent Peer Review?

The Royal Society of Chemistry supports the principles of open science, which include working towards a more open and transparent research culture. Transparent peer review is an important strand of our commitment to open science.

Transparent peer review serves to shed light on the aspects of the journal publication process that are usually hidden. The benefits include:

- Increased transparency in editorial decision-making.
- Readers can learn from the editors' and reviewers' insights, and the published peer reviews can serve as an educational tool for those new to peer review.
- It showcases the hard work and effort of the editors and reviewers in evaluating a manuscript, and of the authors in revising their work in response.
- It may encourage higher-quality and more constructive reviewer comments.

## Is Transparent Peer Review available on all Royal Society of Chemistry journals?

Not yet, but the number of journals offering it is growing. We are actively consulting with our Editorial Boards and journal communities and expect many of our journals to start offering transparent peer review soon.

## Is Transparent Peer Review optional for authors?

Yes, authors opt-in to transparent peer review during the submission process. Authors can change their mind about transparent peer review at any point prior to acceptance.

## Can reviewers opt out of Transparent Peer Review?

By agreeing to review for a journal that offers transparent peer review, reviewers must agree to have their reviewer comments published, if the author selects transparent peer review. Reviewers cannot opt out, unless there are very exceptional circumstances, which should be discussed and agreed with the editor.

## Do reviewers remain anonymous?

Yes.  Reviewers' comments are published anonymously. We take the reviewers' right to remain anonymous very seriously. Reviewers remain anonymous unless they choose otherwise by including their name in their comments to the author.

## What about journals that offer double-anonymised peer review?

Transparent peer review is compatible with both single-anonymised and double-anonymised peer review.  Double-anonymised peer review applies before publication, where the identity and affiliation of the authors is kept anonymous from the reviewers during the peer-review process. Transparent peer review is applied after publication where the associated editor's decision letter, anonymous reviewers' comments and authors' response are published.

## What can I do if I notice that some of the peer review history is missing?

We are currently unable to show comments or responses that were provided as attachments, and we are not yet able to publish the peer review from a previous journal in instances where a manuscript has been transferred. If the peer review history indicates that attachments are available, or if you believe there is review content missing from an article with transparent peer review, you can request the full review record from our Publishing customer services team at RSC1@rsc.org.

## Why can't I see the editor's name on the decision email?

In most cases the full decision email, including the editor's name, is available as part of the peer review record. However, editors do have the option to publish their decision letters anonymously.
